# Target preparation for research with charged projectiles

**DOI:** 10.1007/s10967-013-2652-2

**Published:** 2013-11-19

**Authors:** Anna Stolarz

**Affiliations:** Heavy Ion Laboratory, University of Warsaw, ul. Pasteura 5a, 02-093 Warszawa, Poland

**Keywords:** Vacuum deposition, Rolling, Powder compacting, Radioactive targets, Heat dissipation, Target characterisation, Impurity

## Abstract

The paper reviews in a compact format the techniques most frequently used for target preparation, such as rolling, powder compacting, and vacuum deposition. The survey covers also the techniques used for target characterisation (thickness, purity) and problems related to the extension of target life-time and time of uninterrupted experiments with use of targets.

## Introduction

The term ‘accelerator-based research’ covers all kinds of research performed with accelerated projectiles including not only nuclear physics studies but as well solid state research, radiobiological studies or particle therapy in oncology, just to give some examples. The type of studies determines the target parameters and method of its preparation.

A target used in nuclear research can be defined as an object or system subjected to bombardment by particles such as electrons, protons, etc., or to radiation. The projectiles could originate from spontaneously decaying radioisotopes or can be delivered by an apparatus producing charged projectiles such as elementary particles or heavy ions.

The thin metallic foils applied by Hans Geiger and Ernest Marsden in 1909 for the observation of the behaviour of alpha particles [1], what resulted in a new concept of the atomic structure suggested by Rutherford in 1911 [2], and the tube filled with nitrogen gas used in studies on alpha particles collisions with light atoms carried out by Rutherford and his team in 1919 can be considered as first solid phase and gaseous targets used in nuclear studies. At latter, the first nuclear transmutation was observed when 7.83 MeV *α* particles emitted by ^214^Po were passing through the glass-tube filled with nitrogen gas [3]. In both cases the projectiles originated from spontaneously decaying radioisotopes.

The use of targets for accelerator-based research started with the 1932 experiment by John Douglas Cockcroft and Ernest Thomas Sinton Walton, resulting in the first accelerator induced nuclear transformation [4]. The bombardment of a ^7^Li target with protons of 0.45 MeV delivered by a linear accelerator, known as the Cockcroft–Walton cascade generator, resulted in the production of two alpha particles. The energy of protons delivered by this linear accelerator was sufficient for studies of light nuclei but not for nuclear research with heavier nuclei. To produce projectiles with higher energy using a linear accelerator meant much longer vacuum lines, much more space for accommodation of such devices and problems with maintaining the high voltage. This led to the idea of a construction, where ions are kept in a spiral trajectory and get accelerated consistently by a comparatively low radiofrequency voltage. The first device, a 4.5 inch (~11 cm) cyclotron, was able to boost protons up to 80 keV but it was not satisfying its creators. In quick succession bigger machines were built.

Targets for the first experiments were prepared in a very simple way: by applying contemporary available craft products [1], by filling containers with gas [3], by pressing powders into cavities in backing plates, by drying a solution of the target material on backing plates (Table 1).

**Table 1:** Targets used at experiments on the transmutation of elements by protons (carried out by Oliphant and Rutherford [5] following the first studies performed by Cockroft and Walton [4])

Target		Preparation	Target description, as in [5]
Li	Li_2_O	Burning Li in air and holding a piece of cold steel in the ascending stream of vapour	Thin; film invisible, thickness estimated by stopping power as <1 mm air equivalent “so that when bombarded it is unlikely that any proton made more than one collision with Li nucleus”
B	Na_2_B_4_O_7_·10H_2_O or Na_2_[B_4_O_5_(OH)_4_]·8H_2_O	Evaporating a single drop of a diluted solution of borax as uniformly as possible over the surface of an iron target and then heating to a red in an atmosphere of coal gas	“The weight of borax may be calculated from the strength of the solution and we find that there is sufficient boron present to give uniform layer of about 0.7 molecules (average) thick. This could not have been deposited uniformly but the film was certainly thin enough to assume single collisions only with bombarding protons”
Fe	Iron or mild steel		
O	Oxide	Steel surface oxidised by heating it red hot in air	
Na	Metal	Commercial	
Al	Metal	From Prof Kapitza	
N (NH_4_NO_3_), Be (BeO), F (FeF_2_), Au, Pb, Bi, Ta, U, Th			

In these early experiments it was important just to have a usable target but as it can be seen from the target description given by the authors the homogeneity of the target material distribution was of concern too.

The development of accelerators able to deliver high energy projectiles increased the requirements for the target quality. This triggered the development of techniques used for target preparation to satisfy the demand for targets with more sophisticated properties, robust under bombardment with high energy projectiles. Initially the targets were prepared by the experimenters themselves but with time the target design and preparation became more complex and required wide-ranging skills. To enhance the exchange of the ideas and knowledge on techniques used for target preparation, a seminar gathering the target makers was organised in 1963 at Geel, Belgium, most probably the first of such kind. Further meetings were organised in not always regular intervals, sometimes at only regional level, until 1975, when the International Nuclear Target Development Society (INTDS) was incorporated. The INTDS started organising regular world meetings, initially annual and later biennial, continuing to date [6 and www.intds.org].

## Target preparation

Targets used in nuclear research in most cases are made of the enriched isotopes. They have various dimensions, thickness, physical (solid, liquid, gaseous) and chemical (elemental, compound or alloys) forms. They can be self-supporting or on a backing, they can be used inside the accelerator (so called internal targets), or in external setups on the beam lines.

The majority of the experiments in nuclear physics studies use thin solid state samples and thus most of the attention in this work will be given to the preparation of this type of targets.

Nuclear physics studies usually require targets with an areal density (i.e. thickness) of ~1 μg/cm^2^ up to 10–20 mg/cm^2^. Targets in the thickness range of hundreds of mg/cm^2^ are used for radioisotopes production with accelerators mainly for medical application.

The parameters of a target depend on the experiment but there are requirements which are common. Apart from the requested thickness target should in most cases have a relatively uniform thickness distribution, good mechanical strength and stability under the beam, as well as high chemical purity (the isotopic purity mainly depends on the enrichment of the available material).

It is very difficult to classify the targets by the method to be used for their preparation. The choice of the method depends on many parameters such as the chemical or physical form of the starting material, the type of target (e.g. self-supporting or backed), or the target thickness. This makes it difficult to make any rough indication, which production technique is the most suitable for a specific target.

The method should not only be effective, i.e. assure the production of the final target with the required parameters, but as well should be efficient in terms of minimising the costs, especially when made of very expensive enriched isotopic material. To optimise the usage of this material before producing the final isotopic target the method is developed or tested with natural material. However, it is necessary to consider that the isotopic material may behave differently.

In general the methods applied for target preparation can be roughly classified as mechanical, physical and chemical:

- mechanical reshaping and powder processing: cold rolling, powder compacting (including sedimentation and sintering), HIVIPP (HIgh energy VIbrational Powder Plating).
- physical: vapour condensation at which the deposited material can be heated by resistant heating, electron bombardment, electron beam gun, levitation heating and melting, ion beam sputtering, electrospraying.
- chemical: electrodeposition, thermal cracking, polymerisation.

## Mechanical reshaping

### Rolling

As Frank Karasek wrote in one of his papers “metal rolling—especially the preparation of very thin metal foils—is a great deal more complicated than generally assumed” [7].

Nevertheless, this technique should be considered as one of the primary methods of target preparation, specially for expensive isotopes as the waste of material with this technique is minimal and the method is known for yielding a high quality targets.

In this method the material is reshaped (transformed into foil and/or thinned) using a rolling mill The material to be rolled is placed in a rolling pack (kind of envelope made of stainless steel sheet that is folded over or made of two pieces welded at corners) to protect the material from collecting impurities from the rollers and to facilitate manipulation of the rolled material. The other important reason of using the stainless steel pack is that it allows reaching the foil thicknesses of the order of 1 μm or less as required for targets what due to precision limitations of a standard rolling mill, would be difficult.

Thickness reduction during the rolling process should not be larger than 10–15 % in a single pass at the initial stage and 5–8 % later, but these values are only indicative as they depend on the hardness and malleability of the rolled material. Excessive thickness reduction per pass may cause damage of the crystal structure of the thinned foil leading to its disintegration or may induce its sticking to the protective pack. To attain the good results it is important to keep scrupulous cleanliness during the whole procedure. The foil and the interior of the rolling pack should be free from dust, scratches, and fingerprints to minimize the chance of damaging the prepared foil.

The achievable thickness of the final product varies a lot from material to material depending on its malleability. Generally it is assumed that the lower thickness limit for most of the metals is at the level of ~1 mg/cm^2^ but Mo foils as thin as 130 μg/cm^2^, as reported by E. Kellner and P. Maier-Komor [8], or even as low as 50 μg/cm^2^ reported by Karasek [9] for Mg foils produced by thinning foils prepared by vapour deposition in a high vacuum can be produced with this technique as well. In case of harder metals it is recommended to use interstep annealing [10] for stress relaxation. For best results this should be performed in vacuum or in an inert gas atmosphere [11].

It is much more difficult to achieve a low thickness with soft materials. The practical limit is around 2 mg/cm^2^ but in some cases even 2.5 mg/cm^2^ is achievable only with considerable effort. To facilitate rolling of soft materials and generally to obtain pinhole free foils of low thickness the use of lubricants such as silicon oil is recommended in order to decrease adhesion of the rolled foil to the stainless steel pack. In some cases lining the pack with sheets of acid-free paper or Teflon or other plastic materials is helpful. Rolling soft metals together with other metal as backing, if allowed by ‘physics’, will produce a target of lower thickness than in case of unsupported rolling ([12] and author’s own experience).

The effort of refining the rolling procedure is rewarded by a target with a more robust behaviour under bombardment with heavy ions than foils of the same thickness produced by vacuum evaporation–condensation [8].

Air-sensitive metals should be rolled either in an inert gas atmosphere or coated with a protective layer, which is stable in air and is not expected to interfere with the studied reaction [13].

### Material preparation

In most cases material requires pre-treatment and preparation for rolling. Impurities in the starting material may cause brittleness, pinholes or cracks in the rolled foil. Thus, if possible, the purification of the material by remelting is recommended. In case of materials supplied as oxides or other compounds conversion into a metallic malleable form is required.

#### Melting

In the easiest case when a metal is supplied as grains or in powder form it requires only vacuum melting to produce a rollable bead. Usually, during melting of the material the conceivable impurities collect on the surface of the bead and can be removed mechanically by scratching. Thorough remelting of the material removes the impurities, including oxygen, present in the starting material and results in good quality malleable beads. Even in case of critical materials with respect to rolling such as molybdenum it allows the application of cold rolling from the start [14] because beads are not cracking into small pieces as reported by Karasek [7]. Unfortunately such remelting may cause big material losses.

#### Conversion into metallic form

In case of materials available as compounds the conversion into a ductile metallic form is more complex and may introduce impurities originating from the chemicals used in the process.

When material is in oxide form it could be converted to the metal by metallothermic or by hydrogen reduction.

In a vacuum metallothermic process the reductant and its oxide as well as reduced oxide should have a much lower vapour pressure than the yielded metal to minimise the risk of contamination [15].

For low temperature vacuum reduction of metals with high vapour pressure most frequently La or Zr are used as a reductant, while for high temperature reduction use of Th or Hf is more suitable [16]. This is by no means a complete list of reductants, since Al [17], C [18], Ca [19], CaH_2_ [20], Ta [21] are as well used for this purpose.

For a metallothermic reduction in vacuum the powdered target material and reductant are thoroughly mixed with a stoichiometric excess of 20–50 % of the reductant and compacted to a tablet in a compression mould. The tablet is placed in a crucible which is heated in vacuum to the appropriate temperature. Oxides pre-heating in air at ~1300 K is recommended for a successful reduction [22, 23].

**Fig. 1:**
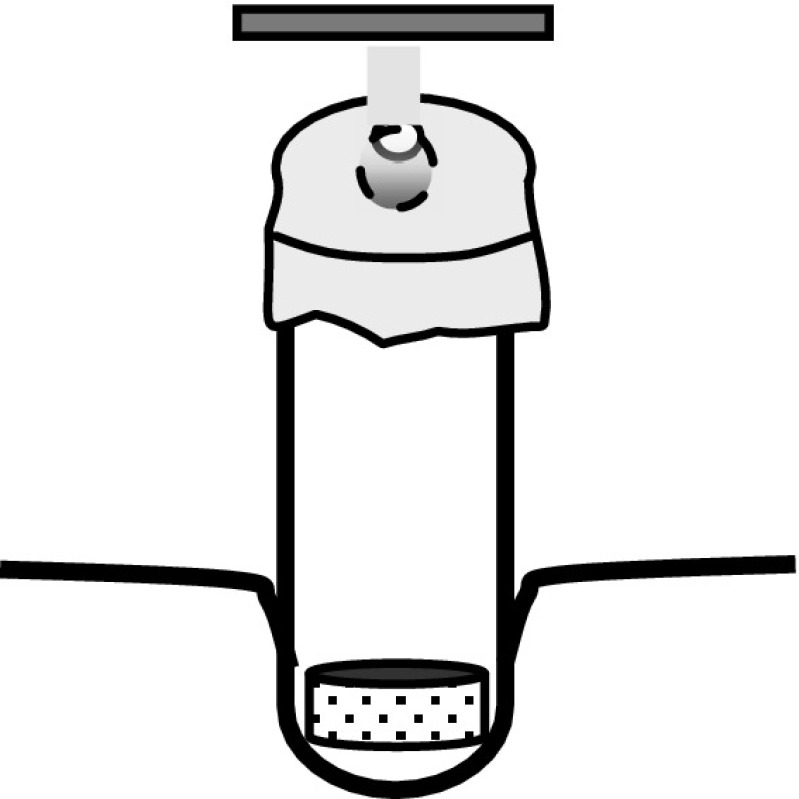
Example of the set-up for the collection of the reduced material as a bead. A tube-shaped Ta crucible is covered with a thin Ta foil with a ~1 mm diam. hole in its *centre*. The foil is fixed to the crucible with a one-loop spring made of Ta wire

The reduced metal can be directly evaporated to produce the target or can be collected in form of a bead for subsequent rolling. To collect the reduced metal a pinhole-crucible (presented in Fig. 8) or a tube-shaped crucible closed with a lid with a small hole should be used. A cooled substrate should be placed above the hole at a distance of ~1 mm to condense the vapour and start the bead growing (Fig. 1).

Reduction with hydrogen. The method described above is very practical for the reduction of alkaline earth and rare earth elements, but for metals such as Zn or Cd or Pb the reduction in a hydrogen atmosphere is more advantageous. By heating in a tube-furnace in a hydrogen atmosphere the oxides Cr_2_O_3_, Tl_2_O_3_, Fe_2_O_3_, CuO, GeO_2_, In_2_O_3_, PbO, Sn_2_O_3_ and WO_3_ were reported [10, 24, 25, 47] to be reduced to the metal form with an efficiency close to 100 %. The hydrogen reduction can be carried as well in a standard vacuum evaporator. Reduction in the evaporator allows direct use of the obtained metal for evaporation–condensation (see Sect. “Melting”) omitting exposure of the prepared metal to air when loading to the evaporator.

The reduction with hydrogen is a very simple method requiring only precautions to supply very pure hydrogen, free of humidity and oxygen. In case of metals with high affinity to oxygen the hydrogen should be additionally purified by passing through a palladium diffusion cell [24]. The method assures a final product virtually free of contamination.

The conversion of compounds other than oxide into the metallic form often requires pre transformation into oxide otherwise the final product can be contaminated with products of a side reaction. For example in direct reduction of barium carbonate BaCO_3_ the reduction side product, carbon dioxide CO_2_, reacts with just produced Ba giving barium carbide BaC_2_ and barium oxide BaO In such cases the earlier conversion of the starting material into oxide that is used for further reduction eliminates those side reactions and thus the production of contaminants [26].

## Powder processing

### Tablet pressing

**Fig. 2:**
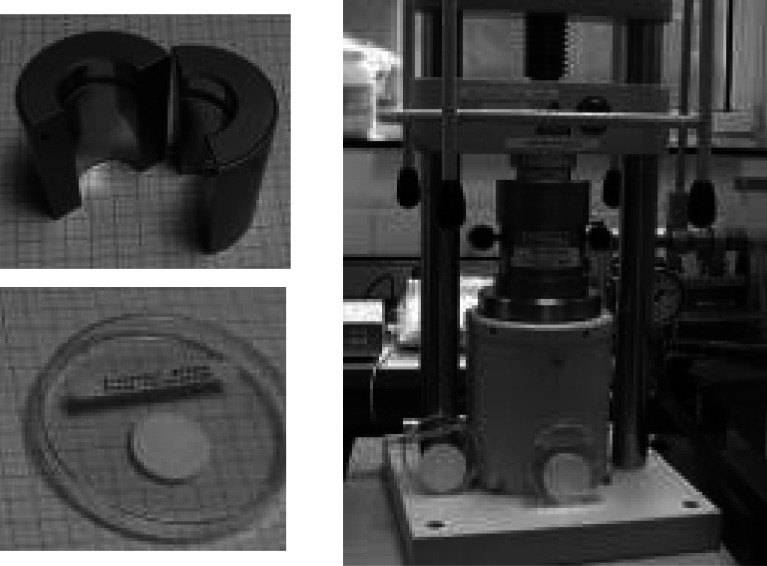
An S target pellet (*left*) prepared by powder pressing with use of a hydraulic press (*right*)

The method is recommended for relatively thick targets (~20 mg/cm^2^). Hereby, a target can be prepared as a self-supporting tablet (Fig. 2) or can be compressed into a container.

The powder pressing should be carried with simultaneous pumping to reduce the amount of gas (air) that may be compressed in the tablet. The excess of the gas in the tablet may cause its explosion when heated by the beam or when heated in crucible for evaporation.

**Fig. 3:**
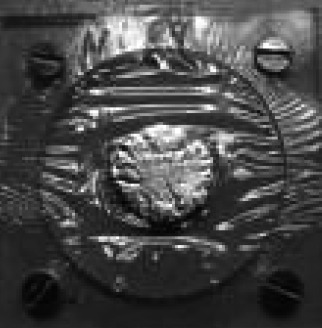
Ni target of ~72.1 mg/cm^2^ prepared within 1 h. Grains of Ni pressed into a 95 mg/cm^2^ pellet of 5 mm diameter were rolled to produce a target of required thickness and diameter (10 mm). The target was used in neutron halo studies [27, 28]

The preparation of a tablet is relatively quick so it can be used as well as an intermediate step in the production of targets of material the melting of which is difficult and time consuming e.g. Ni. Figure 3 shows the thick target prepared by combination of two techniques: tablet pressing followed by rolling.

In case of very hard materials that can not be pressed into a tablet the addition of a binder (graphite, soft metal, organic binder) can be a solution, if acceptable by the experiment.

**Fig. 4:**
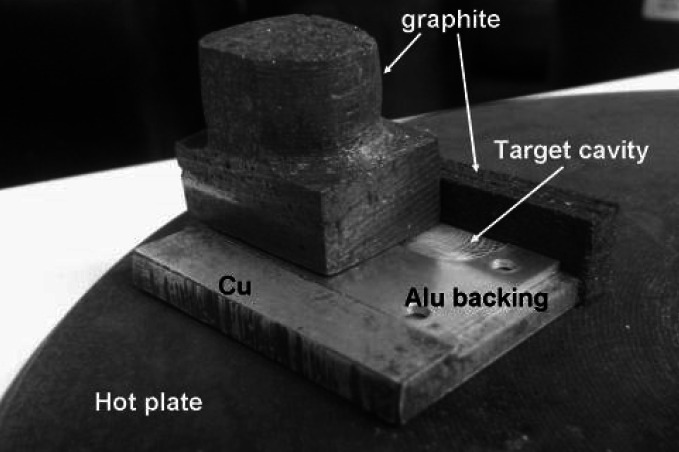
Tool for preparation of Bi layers on an Al backing by melting Bi metal powder on a hot plate and distributing the melted material along the target cavity with simultaneous pressing when hot [29]

For some materials hot pressing or sintering (Fig. 4) may be advised to assure the appropriate mechanical strength of the final product. Such procedures are frequently applied in case of targets for medical radioisotopes production when a relatively thick target, which is able to withstand the bombardment by an intensive beam, is required.

### Sedimentation

Precipitation is a useful method in the case of metals of high melting point and low vapour pressure or compounds which are difficult to handle by the usual vacuum evaporation technique, particularly if concerned with enriched isotopes available only in minute amount [30].

The powdered samples after suspending in a suitable liquid are precipitated on a substrate mounted at the bottom of the precipitation vessel. By this procedure targets can be prepared on a backing or as quasi self-supporting if a binder is included in the target body.

#### Spontaneous/gravimetrical

Targets of B, U and Pu requested for works on the Manhattan project were prepared by drying drops of liquid containing a suspended fine powder of a target material as reported by Dodson [31] in a series of documents reporting studies done during and just after WWII. Addition of polymers such as polystyrene [32] allowed the preparation of the ‘self-supporting’ targets by depositing the slurry on a glass plate and releasing them when dry.

**Fig. 5:**
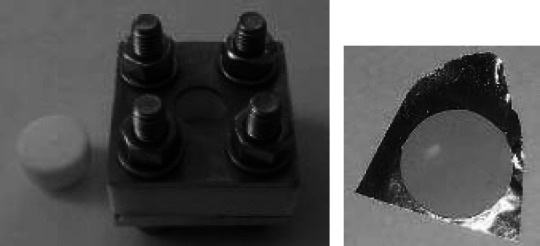
Vessel used for preparation of targets by gravimetrical sedimentation (*left*) and a 3 mg/cm^2^ Nb_3_O_2_ layer on an Au backing, deposited by this method from a suspension in diluted epoxy resin

Using the sedimentation technique, robust and mechanically stable targets of any shape and size, specified by the inner dimension of the vessel at which the precipitation is conducted, can be prepared (Fig. 5). The slurry of the fine ground target material in the organic binder placed in the well levelled vessel should be left undisturbed in a dust free hood till the solvent is evaporated [33]. The temptation of speeding the evaporation by warming the slurry should be avoided as convection causes whirls that may negatively influence the flatness and/or thickness uniformity of the sediment.

#### Imposed/centrifugal

This modification of the target production by the sedimentation was introduced by I. Sugai [34, 35] who used a centrifuge to precipitate powder suspended in paraffin.

The big advantage of the sedimentation methods is their high yield which can be above 95 %. In addition these methods are very simple and quick.

### HIVIPP

The method of HIgh energy Vibrational Powder Plating allows the preparation of targets with areal densities ranging from μg/cm^2^ up to mg/cm^2^ with a very high yield and of excellent thickness homogeneity.

The method is based on the motion of microparticles of the material in an electric field. The deposition process is carried in a vacuum in a vessel with electrodes mounted at its top and at the bottom. A high voltage (>2 kV) applied to the electrodes causes the particle motion towards the electrode of the opposite charge on which they are deposited.

In 1996 I. Sugai, the method inventor, reported [36] preparation of various targets with thickness ranging from 50 μg/cm^2^ (for natB) up to 2 mg/cm^2^ for Cr with this method.

The method can be applied to prepare not only supported but as well self-supporting targets, thin as mentioned above 50 μg/cm^2^ of B or thick as e.g. 90 mg/cm^2^ Si targets [37]. The method is as well suitable for preparation of plunger targets as was demonstrated by a group from Stony Brook [38] who produced the target of ^11^B on ^93^Nb.

Comparison of yield and thickness homogeneity of targets made by evaporation and by HIVIPP method shows the superiority of the last one [39].

Unfortunately there is a disadvantage of this method, especially essential for thin deposits. The target layer can be contaminated by the backing material due to sputtering induced by the accelerated powder grains [37]. Another drawback is that the preparation time is very long. It often takes several hours to achieve a thickness of a few mg/cm^2^.

## Vacuum deposition

### Vacuum thermal deposition: evaporation–condensation

**Fig. 6:**
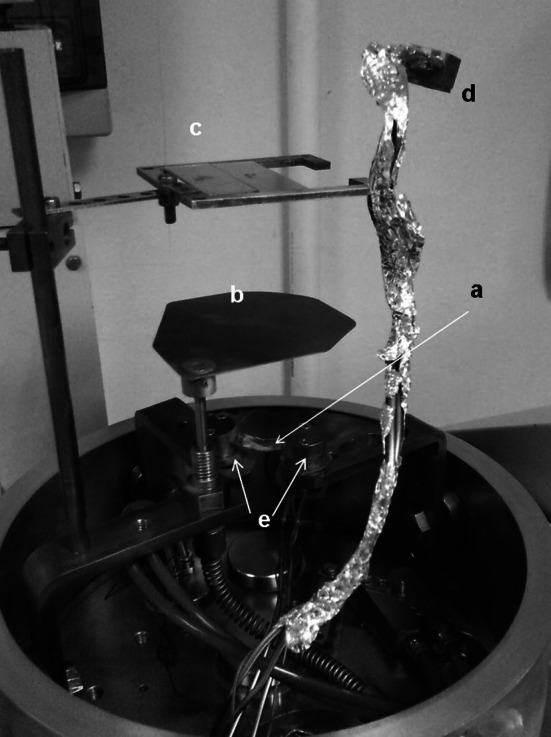
Typical set-up for evaporation in the high vacuum showing the vapour source (*a*) (in the photo a crucible for resistant heating mounted between water-cooled electrodes (*e*)), the movable shutter (*b*) (used to collect initial vapour with potential impurities and to stop the deposition at the desired moment), the substrate holder on which the vapour condenses (*c*) and the sensor head of the quartz thickness monitor (*d*). The visible aluminium wrapping protects the parts of the set-up against build up of deposits

Target preparation by evaporation–condensation is carried in a high vacuum. The source material is evaporated from a heated suitable crucible and collected on a substrate placed above the vapour source. Volatile impurities present in the source material are removed prior to evaporation–condensation by heating the material at temperature close to, but lower than, its melting point. The substrate is protected against those impurities by a movable shutter placed between vapour source and substrate. A typical set-up for vapour deposition in vacuum is presented in Fig. 6.

Typically relatively thin, in the range of up to a few hundreds of μg/cm^2^, both self-supporting and backing supported targets are prepared with this method but deposits in range of mg/cm^2^ can be produced as well [40] when needed. The method allows production of layers with homogeneous material distribution over relatively large areas, however at the price of a very low collection efficiency, therefore, a big material loss.

**Fig. 7:**
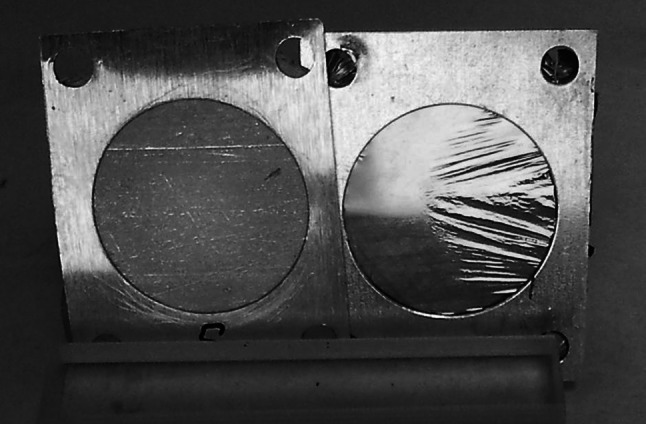
Self-supporting Cu foils prepared by vapour deposition on the parting agents: Betaine (*left*) and Lensodel (*right*)

The quality of the deposits depends on such process parameters as deposition rate, substrate temperature [41–43], properties of the evaporated material, way of material heating, etc. [44]. It depends as well on the type of the parting agent used when self-supporting foils are aimed (Fig. 7 and Sect. “Parting agents used in self-supporting targets preparation”).

Material condensation on a relatively cold substrate produces inhomogeneous films consisting of small islands which do not merge together. Thus, if a relatively very thin layer is produced, it will break up when released from the substrate. Nevertheless, substrate cooling can be applied when vapour condensation has to be enhanced [45] to increase the efficiency of the target preparation. Condensation of Zn and Cd isotopes, materials which condense poorly, was enforced by Maier et al. [21] applying a LN_2_-cooled ceramic substrate.

If the substrate is heated during deposition, the ‘islands flow’ during condensation and thus may produce a continuous layer. Substrate heating allows preparation of targets with special characteristics like e.g. magnetic properties of gadolinium which can be achieved only when the deposit crystallises at an appropriate temperature [46]. Substrate heating allowed Gursky et al. [41] to prepare thin self-supporting Ni and Pt foils of 40 and 80 μg/cm^2^, respectively.

#### Way of heating the evaporant

##### Resistant heating

**Fig. 8:**
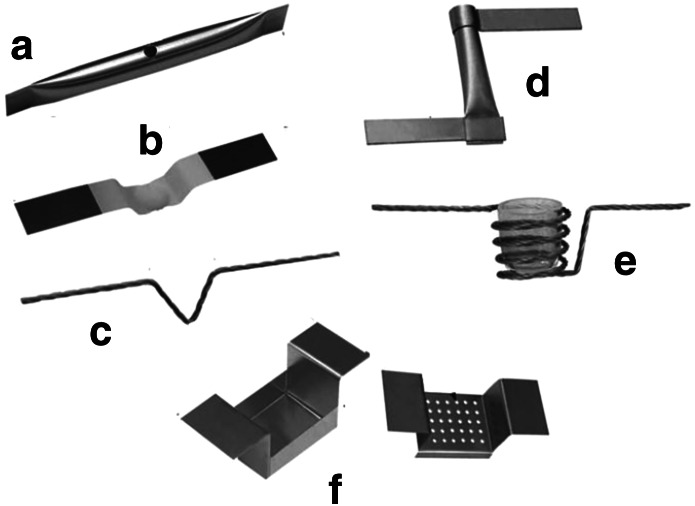
Examples of vapour sources for resistant heating: **a** pinhole, **b** open boat with Al_2_O_3_ protective layer for evaporation of alloying materials, **c** loop source, **d** tube-type, **e** crucible with basket heater, **f** with cover (e.g. for powder evaporation)

Materials with a melting point lower than 1850 K can be evaporated by electrical resistant heating from boat- or tube-shaped crucibles (Fig. 8) placed between water-cooled electrodes (Fig. 6).

A boat-shaped crucible covers a deposition angle of ~2π so when wastage of the evaporant has to be minimised the tube-shaped is advised. The higher the ratio of the tube length to the tube diameter the better the vapour flux is collimated thus decreasing the angle of vapour distribution.

For materials with high vapour pressure, such as Zn, Cd, Ca or Mg, which do not readily condense on a substrate unless vapour stream density is extremely high, the pinhole crucible (Fig. 8a) is recommended [47] if condensation can not be enhanced by substrate cooling. As mentioned above, condensation of the vapour of these materials can be greatly enhanced by depositing on a cold substrate and thus, the tube-shaped or even open boat crucibles can be applied as well preserving a good condensation efficiency.

Not only the shape but as well the crucible material compatibility with the evaporant must be considered when choosing the crucible. The crucible’s melting point has to be much higher than that of the evaporated material, otherwise a high amount of impurities will be introduced into the target deposit. Boats made of the refractive metals Ta, Mo, W [48] are mainly used in resistant heating. Resistant heating can as well be applied to evaporate the evaporant placed directly between the electrodes. Good quality carbon foils with various thicknesses are prepared this way [49, 50] (more about carbon foil production in Sect. “Stripper targets”).

##### Electron bombardment and electron beam gun heating

For materials with a higher melting point electron bombardment [51] or electron beam gun heating is recommended [52]. The latter is especially suitable for materials alloying or chemically reacting with hot crucible materials causing the contamination of the deposit.

The two terms, electron bombardment and electron beam gun heating, are often mixed up when technologies used for heating of evaporant are described.

**Fig. 9:**
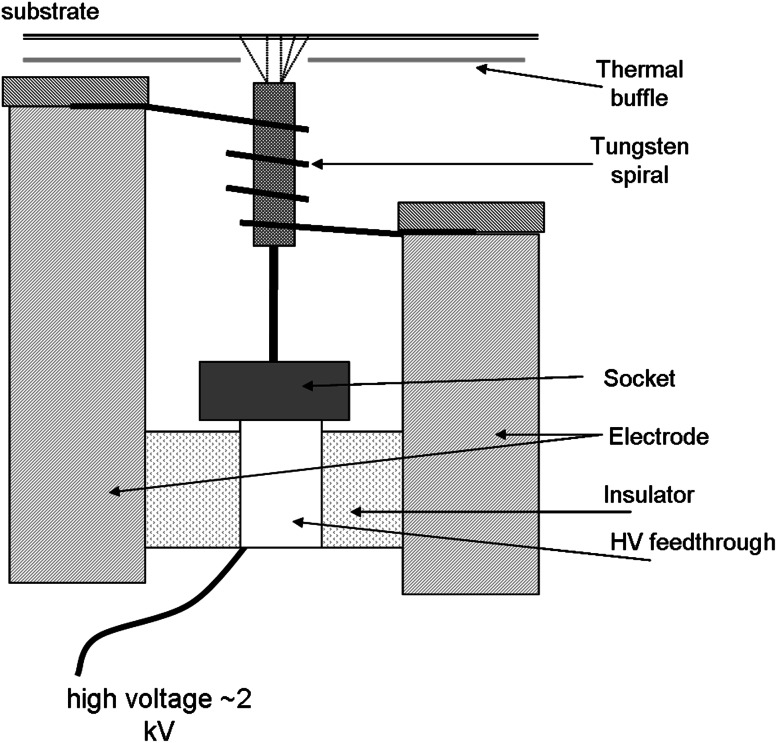
The electron bombardment set-up—drawing based on the scheme presented in [54]

In electron bombardment the crucible is heated by electrons emitted from a tungsten spiral surrounding a tube-shaped crucible and accelerated by a high voltage applied to the crucible [53]. Figure 9 shows schematically the set-up proposed by Westgaard and Bjørnholm [54] for this type of heating.

To save expensive isotopic material narrow tube-shaped tantalum or graphite crucibles are used for evaporation. For material incompatible with the crucible boron nitride or zirconia liners are recommended [47].

**Fig. 10:**
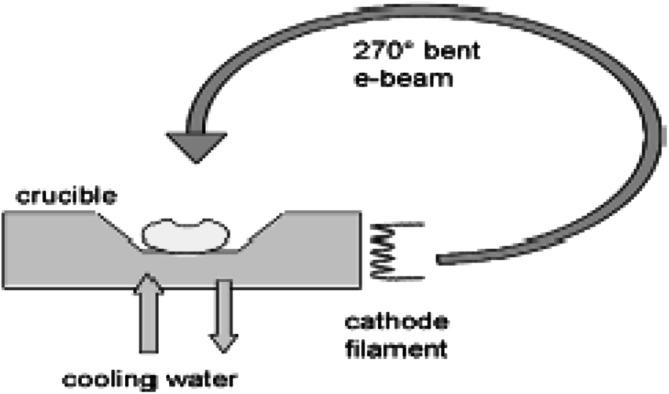
Scheme of an electron beam gun with 270° bent beam directed into the crucible by a magnetic field

In the electron beam gun heating method the evaporated material is placed in a water-cooled crucible and is heated by a focused beam of electrons emitted from a filament. Nowadays 270° electron beam bended devices (Fig. 10) are in use in order to avoid contamination of the filament by the evaporated material. This way of evaporant heating is suitable not only for materials with a high melting point or low vapour pressure [55], but as well for those which tend to alloy with hot crucible material. In this way of evaporant heating, the hearth of the electron beam gun is water-cooled and thus limited to a temperature lower than 100 °C. Moreover, in case of relatively large quantity of evaporant material and a well focused electron beam, the evaporant is melted only at the area of the beam spot, while the rest remains solid.

Extensive studies of the influence of the beam energy, beam size, etc. on the quality of targets produced by electron beam gun heating were presented by Maier-Komor in [56].

### Vacuum cold deposition: sputtering

Sputtering process is the ejection of material from the surface of solids at bombardment with energetic particles such as noble gas ions accelerated in an electric field. The ejected material is collected on a substrate producing a thin layer. The deposition of the target material is independent of its vapour pressure and is very economical.

This technique of material deposition eliminates problems mentioned above such as target contamination by the crucible material when the vapour pressure of the deposited material and the crucible are comparable i.e. in case of making targets of refractory metals.

It offers as well the possibility of relatively easy preparation of a pure targets of metals that alloy with the common evaporation crucibles i.e. made of Mo, Ta and W.

Although there is no contamination from the crucible, the contamination by the noble gas used for sputtering is possible. But this can be turned into advantage when ‘gaseous’ targets have to be prepared [57, 58] (see Sect. “Gas targets”).

**Fig. 11:**
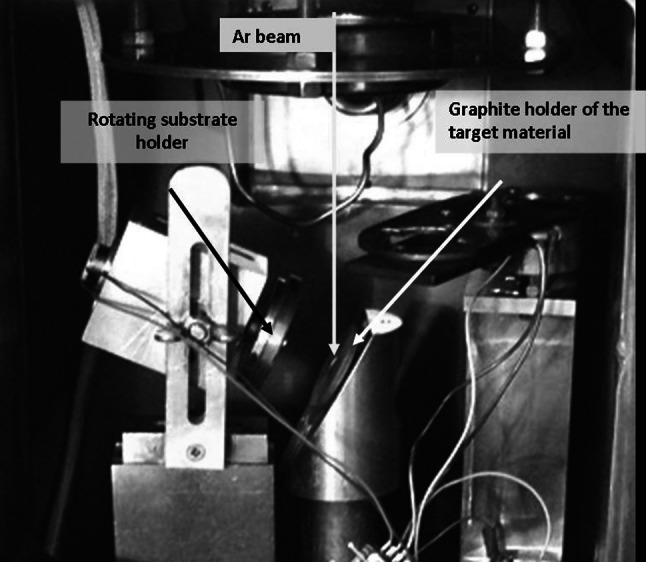
Heart of the sputtering device at Target laboratory, Department of Physics, University of Jyväskylä, Finland

In 1971 Sletten and Knudsen [59] reported the construction and application of a compact sputtering device with focused ion beam allowing production of targets from minutes amounts of material (Fig. 11). The sputtering efficiency was compared to that of electron bombardment and was reported to be similar (~25–30 % collection at the distance of 4 cm in both set-ups).

According to Baumann and Wirth [60] and Maier [61] the quality of foils produced by sputtering is better than of those made by evaporation.

### Deposition rate and deposit thickness monitoring

The preparation of foils by vapour deposition in vacuum often requires monitoring the deposition rate as well as the thickness of the growing deposit. A method widely used for it is based on the change of the vibration frequency of a piezo-electric crystal with the change of its mass.

The quantitative relationship between change of crystal mass and frequency shift was established by Sauerbrey [62] showing that for thin films deposited on a piezo–electric crystal the change (decrease) of the crystal vibration frequency d*f* is proportional to a change of mass *dm*:

$$ {\text{dm }} = \, - {\text{C}}_{\text{Q}} \cdot {\text{d}}f $$

where C_Q_ is a coefficient proportional to crystal active area, its vibration frequency constant and its density and inversely proportional to the square root of crystal frequency at start of deposition. Behrandt and Love [63] found that frequency shifts due to temperature variations of the crystal can be reduced using a cooled holder and reported the construction of a device monitoring the deposition rate. In 1964 Muggeleton and Howe [64] reported the construction of the set-up able to monitor the thickness of a deposited film and the rate of the process. In the following the Quartz Crystal Thickness Monitor has been improved to eliminate measurement errors due to temperature effects and mechanical shocks and became widely used in thin film deposition processes. However, its thickness reading should be considered only as a first approximation and the target thickness should be determined outside the deposition chamber by another method. Nevertheless there are no doubts that vapour deposition without it would be far more complicated. The measurements range of the QCTM is between 1 μg/cm^2^ and several mg/cm^2^ [65].

### Thickness homogeneity: How to achieve?

The distribution of the material which grows on a substrate depends on the distribution of vapour stream emerging from vapour source. The thickness of the deposited material is dependent on vapour stream intensity and may vary distinctly between the centre and the border of the deposit. A common approach for a small surface source is expressed by

$$ {{\varphi }}\left( \alpha \right) = {{\varphi }}_{0} \cos^{\text{n}} \alpha $$

where φ(*α*) is the vapour stream distribution at angle *α* i.e. between the perpendicular *α* = 0 (φ_0_) and the propagation direction, and 1 ≤ n ≤ 4 [66], its value has to be determined experimentally.

**Fig. 12:**
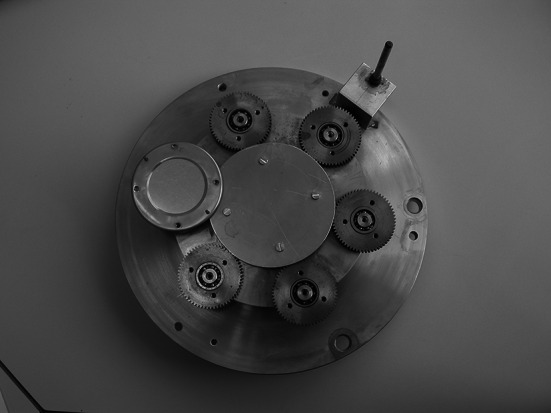
The photo shows the rotator used recently at IRMM (Institute for Reference Materials and Measurements)

The thickness homogeneity of the deposit can be improved by increasing the distance between the source and the substrate. This is, however, bought dearly by a decrease of the deposition yield. An improvement (decrease) of the material consumption is for instance possible by preparing a set of several targets at one run, profiting in addition from a considerable time saving. The material distribution over the relatively big area i.e. on multiple substrates can be rectified by mounting the substrates on rotating plates instead of placing them in fixed positions above the vapour source. This results in a considerable improvement of the deposit homogeneity, as reported by Povelites [67] for production of U and Th targets. A planetary gear driven by a central pinion was composed of substrate holders rotating at their own axis (Fig. 12). A comparison of the thickness uniformity of targets prepared at the same source-substrate distance showed that thickness deviation for the single centrally mounted target and for a set of targets made with the planetary gear, where substrates were mounted at substantial distance from the centre, are comparable.

A comprehensive discussion on the preparation of targets with extremely high homogeneity was given by Maier [68].

An inventive approach to minimize the consumption of source material and working at an extremely short vapour source-substrate distance while achieving a good thickness homogeneity of the deposit on the substrate was reported by Reynolds [69]. He simulated a multi point evaporation source by placing a mesh at the mid-height inside an evaporation tube. It was reported, that 90 % of the source material was recovered and the thickness non-uniformity was at the level of 10 % over an area of ~1 cm^2^.

**Fig. 13:**
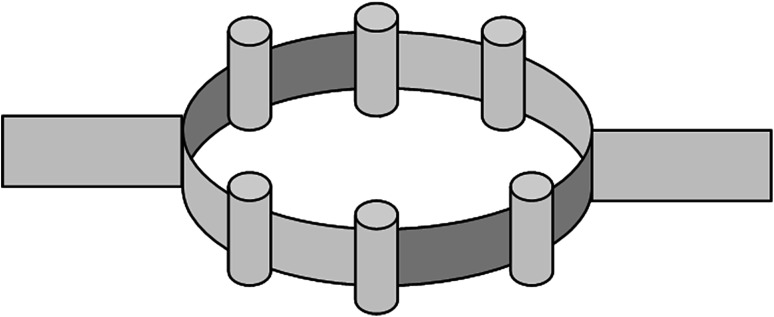
Ring of multiple source crucibles used for the production of deposits with high thickness uniformity when evaporating at a short distance

Another setup allowing production of deposits with good thickness homogeneity when evaporating at a short source-substrate distance was a ring of source crucibles (Fig. 13) used at AWRE (Atomic Weapons Research Establishment, recently AWE) in earlier days [private communication].

### Backing materials

Backings are used to support the thin and fragile target films permanently or only to facilitate the mounting step and in any case should not interfere with the studied phenomena. The most common backings are carbon foils which, although not very strong, are resistant to high temperature and assure good thermal and electrical conductivity. As this is quite common that thin deposits of C are anyhow building up on the target during an experiment (cracked pump oil, 3–5 μg/cm^2^ h [47]) the presence of carbon backings does not introduce additional side-effects to the measurements, what makes carbon foils very popular as backings. However, in view of the mechanical weakness of C-foils other backings such as plastics and very thin metal foils are often considered.

It is impossible to list all current or potential backing materials apart from the already mentioned carbon foils, but the following list presents materials which are most frequently used as backing for targets:

- metal foils: Al, Cu, Au, Ni, Ta
- plastics: collodion ([C_6_H_7_(NO_2_)_3_O_5_]_n_), formvar ([C_5_H_8_O_2_]_n_), Mylar (polyethylene terephthalate ([C_10_H_8_O_4_]_n_), Nylon (polyamide [NH–(CH_2_)_5_–CO]_n_ made from ε-Caprolactam), VYNS (vinyl resin ([C_6_H_9_O_2_Cl]_n_) and polyimide ([C_22_H_10_N_2_O_4_]_n_).

The procedure of in situ production of the last one [70] allows preparation of the foils as thin as 10 μg/cm^2^. With their relatively high temperature resistance [71] they seem to be the best among the plastic materials used as backings [72]). The high quality of polyimide foils was proven by applying it as a backing for thin Ni targets prepared by sputter deposition [73].

Apart from target preparation foils found several other applications e.g. due to their mechanical properties for the production of optical filters applied in astrophysics studies [71] or, because of their relatively high resistance to radiation as a barrier for fission products of radioactive materials such as ^252^Cf used at standard neutron sources [74].

### Parting agents used in self-supporting targets preparation

The choice of the parting agent (e.g. soluble in water or in organic solvent), needed when self-supporting targets are aimed, depends on the chemical properties of the deposited material, on its crystalline structure, and on the required smoothness and thickness uniformity of the produced foil. Extended studies of the parting agents were presented by Braski [75].

Typical water soluble parting agents are inorganic salts such as chlorides, fluorides, a mixture of betaine monohydrate with saccharose which was brought into use by Hermann Wirth and made widely known by Maier-Komor [49], as well as biodegradable fragrance free detergents (Teepol, Lensodel) just to mention a few examples.

In case that the deposited material is water sensitive, an organic solvent has to be used. In such situation for instance compounds like bedacryl (soluble in xylene), acetylamine (hexane), boric acid (glycerine, ether,) formvar (chloroform, toluene), and collodion (alcohol, ether) serve as parting agents.

### Mounting the evaporated target

**Fig. 14:**
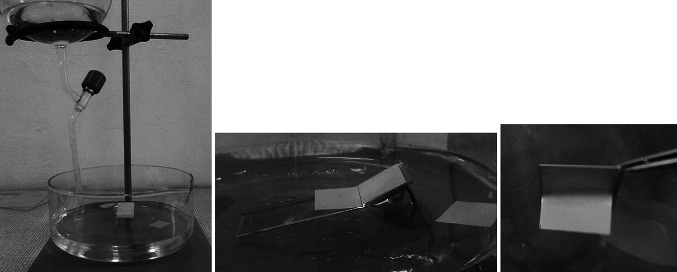
Floating process used for releasing foils from the substrate and fishing on a frame

The substrate with the film of condensed target material should be inserted into a vessel with a solvent appropriate to the compound used as the parting agent. It is recommended to place the substrate plate on a support assuring a ~30–35° angle between the solvent surface and the substrate (Fig. 14). The releasing of the deposited target film from substrate should proceed by raising the solvent level at an appropriate speed. The solvent level can be raised by slow immersing the substrate into the vessel by hand or by raising the vessel while the substrate remains in a fixed position or by raising the solvent level by filling the vessel with the solvent, preferably by adding the solvent through the tube/funnel with its end placed close to the vessel bottom. The latter is recommended by the author as it protects the foils from shock or rugged immersion of the substrate into solvent by hand shake or the elevator vibrations. In case of thin large size foils even the direction of the solvent filling into the floating vessel has influence on the foil survival during the floating process [76].

When a foil is released from the substrate it can be mounted on a frame by fishing it from the solvent surface. The frame should be immersed perpendicularly to the foil and the solvent surface and kept in this position when lifting up the foil to minimise the impact of the solvent surface tension. When dry the self-adhesive forces are sufficient to hold the foil in place in case of thin foils, but for thicker ones the application of an appropriate glue or clamping the foil between two frames may be needed.

The deposit can be as well released from the backing with a sharp razor blade [47, 77] but there is a danger to fracture the deposit during releasing.

## Electrodeposition

To produce a target with this technique the material is deposited on a conductive backing from an aqueous or organic medium [78]. The backing, when needed and possible, by etching [79]. Although electrodeposition technique assures high efficiency and ability to produce targets from a small amount of material and can be applied to many metallic elements [80–84] it is nowadays mainly used for the preparation of radioactive targets [85–94]. Method assures no cross contamination as each deposition cell is devoted to a particular isotope. An important drawback of the method is the possibility of introducing impurities from the solvent or the electrodes.

**Table:** Comparison of the target preparation methods

Method	Thickness limitation	Comments	Efficiency
Rolling	0.5 mg/cm^2^ — >g/cm^2^ soft metals >1.5 mg/cm^2^	Material has to be malleable, often material preparation (compound transformation) before rolling needed what could be the source of impurities	~90 %
Tablet	~20 mg/cm^2^— >g/cm^2^	Excluded for very hard materials unless addition of binder is accepted	>95 %
Sedimentation	Few mg/cm^2^ — >g/cm^2^	Additional material—binder	95–98 %
Electro-deposition	μg/cm^2^ — ~2 mg/cm^2^	Deposited always on a backing but self-supporting feasible by etching the backing, often contaminated by elements present in the used chemicals, max thickness limited due to material pealing	80–90 %
HIVIPP	μg/cm^2^ — mg/cm^2^	Self-supporting or on a backing, applicable to the most materials	95–98 %
High vacuum evaporation	μg/cm^2^ — mg/cm^2^	Self-supporting or on a backing various ways of evaporant heating	2–10 % (could be much higher if deposition performed at close distance of substrate and vapour source)
Sputtering	μg/cm^2^ — mg/cm^2^	Suitable to refractory metal, materials with low vapour pressure, alloying with crucible metals or decomposing at elevated temp., target free of contaminants except ions used for sputtering	20–30 % efficiency,

## Target characterisation

The most important parameters to be estimated for a completed target are its thickness, the thickness uniformity and the identification of impurities.

The thickness of a target is most commonly described as its weight per area i.e. g–mg–μg/cm^2^ and is known as areal density.

The number of nuclear reactions of interest that occur in a unit of time under the bombardment by a beam of projectiles is proportional to the reaction cross-section reflecting the reaction probability, to the beam intensity and to the number of nuclei ‘seen’ by the beam [95].

Since the number of atoms/nuclei in the sample is proportional to its mass it is more convenient to describe the target thickness in areal density unit g–mg–μg/cm^2^.

In early reports on nuclear studies the thickness of the target was expressed in linear units or described as equivalent of the air thickness [1–3] or simply as the mass of the substance used for target preparation but later the areal density units were introduced. Very probably it was related to the introduction of the term of cross section *σ* (1 b = 10^−24^ cm^2^) expressing the probability of the reaction occurrence.

### Thickness and its homogeneity

The most straightforward and oldest method of the target thickness estimation is based on gravimetry, i.e. the weighing a defined area of the target. The accuracy of the method is limited by several factors such as the balance precision, the sample mass stability (especially after sample preparation in vacuum), the stability of the weighing room conditions, etc. Another drawback of the method is that the measured mass is the total mass which gives the average target thickness regardless of the thickness inhomogeneity. There are common concerns about the weighing precision but another significant drawback of the method are as well the errors in determination of the area of the weighed target.

**Fig. 15:**
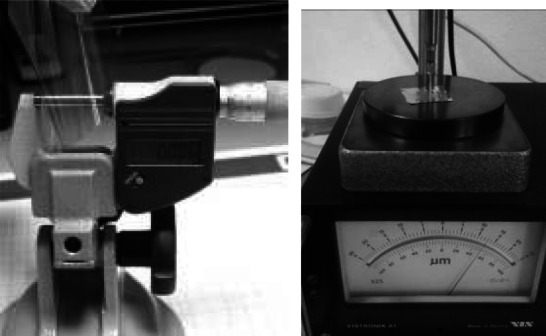
Micrometric screw (*left*) and electrical thickness gauge based on magnetic induction (*right*)

Mechanical or electrical methods (Fig. 15) of thickness determination are less suitable for evaporated targets. This is not only because of the mechanical fragility of measured samples but mainly due to the fact that evaporated films in most cases do not have the same density as the raw material, which is assumed at conversion of the measured linear values into areal density or in calculation of the number of atoms to estimate the number of studied reactions.

The thickness of relatively very thin foils can be estimated spectrophotometrically by measuring their light transmission or absorption [96, 97].

#### Alpha particle energy loss

**Fig. 16:**
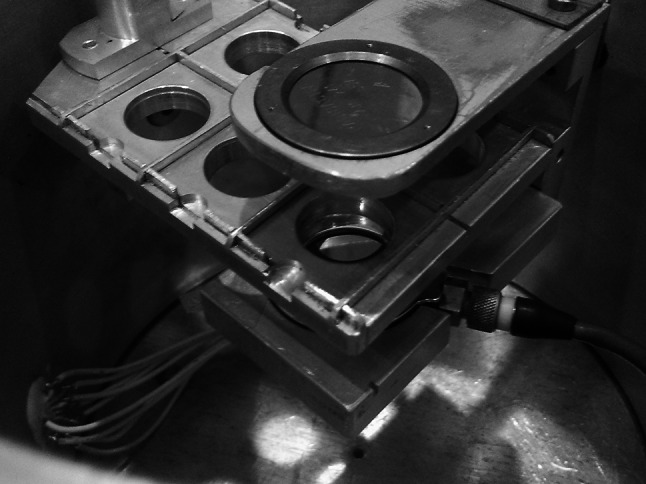
Set-up for the measurement of alpha particle energy loss: the targets are placed in a vacuum chamber on x-movable table while the alpha particles source and detector are on y-movable arm. Such set-up allows target scan without chamber opening

The method is based on the energy loss of alpha particle passing the material (Fig. 16). The areal density for the thin foils can be determined from the relation between the energy loss and the stopping power of the alpha particle for the given energy in the target material

$$ th \, = {{\Updelta}}E/S\left( E \right) $$

where *th* is the measured thickness, Δ*E* is the measured energy loss [MeV], *S* is the stopping power of the material at the energy *E* of the alpha particle [MeV/cm^2^/g].

Since *S* is energy dependent, in the case of the thick foils the total energy loss has to be split [98] into n steps and the thickness is expressed as:

$$ th \, = \Updelta E/n\sum\limits_{j = 1}^{n} {\frac{1}{S(E - (j - 1)\Updelta E/n)}} $$

where *n* is a number of steps with even energy loss.

The upper limit of the method is considered somewhere around the 5 mg/cm^2^. The lower limit of the method depends on the sensitivity of the used detectors, i.e. on the precision of the measurability of the alpha energy peak shift.

The method is relatively simple and can be applied to all materials with known stopping power values. These are nowadays easily available from the TRIM or SRIM programmes.

The method may perfectly serve for estimation of the thickness homogeneity by scanning the target area with a collimated beam of alpha particles [45].

Beta particle [99, 100] as well as X-ray attenuation [33, 101] can be used for thickness and its homogeneity determination for foils in the range of a few to hundreds of mg/cm^2^.

### Impurity analyses

Analyses of the purity of targets are especially needed when assessing newly developed production processes. It is important to estimate the kind and level of impurities, particularly if they are competitive to the studied phenomena.

The analyses can be destructive or non destructive to the sample. Neutron activation analyses (NAA) and Inductively Coupled Plasma Mass Spectrometry (ICP-MS) are very useful when evaluating the new process developed with natural material but in case of final isotopic samples destructive methods can not be considered (mainly due to the costs of the isotopic material). In such case the analyses using the PIXE (proton induced X-ray emission) or RBS (Rutherford back scattering) method are recommended.

In PIXE method the characteristic X-rays of the element are generated by target bombardment with a beam of protons of few MeV energy [102–104] and measured with (Si(Li) detectors [105–107]. Higher energy of protons allows use of the protons beam of submicron size [108].

**Fig. 17:**
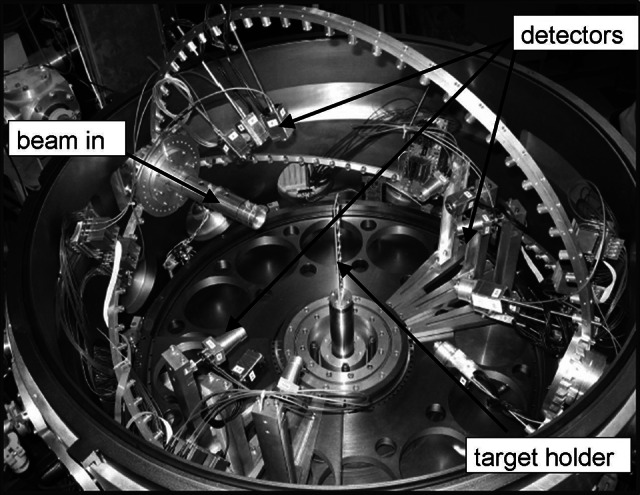
Backscattering chamber with set of detectors placed at various angles. On a photo is a 1 m diameter ICARE multidetector array consisting of Si+CsI telescopes installed at HIL UW (ICARE: Identificateur de Charges A Rendement Eleve)

In RBS method target is bombarded with particles or light ions (p, *α*, ^6,7^Li, etc.) with energy of a few MeV (below the Coulomb barrier). The scattered projectiles are registered by detectors placed at various angles to the beam (Fig. 17). Method allows not only the determination of the impurity and its concentration [104, 106] but as well can be used for sample thickness measurements [109].

## Radioactive targets

The increased availability of isotopes of U, Pu, Th, Am, etc. raised the demand for targets consisting of these radioactive materials. The methods used for their preparation do not differ from methods applied in preparation of stable targets. Some of them like electrodeposition, electrospraying or molecular plating are more popular due to the low costs of the equipment required for target preparation. This allows a permanent allocation of a labware to a specific isotope, thus avoiding cross contamination. Vacuum evaporation, on the contrary, is a very uneconomic method to be used for radioactive target preparation. This is not only due to the low efficiency of the process but due to the requirement of devoting a separate vacuum set-up for each individual isotope to avoid cross contamination. Nevertheless, vacuum evaporation is applied if microscopically smooth targets on thin backings are required e.g. for high resolution nuclear spectroscopy [110]. The high cost of the equipment limits the number of labs being able to afford it. Unfortunately the hot-lab facility which went into service in 1997 at the LMU Munich [111, 112] was shut down in 2011.

Another method resulting in high quality final products such as alloys of radioactive metals, used only in a few laboratories, is levitation heating [113]. The advantages of this method as described by van Audenhove et al. [114] are:

- the material is melted without getting in contact with a crucible, so there is no risk of contamination from the crucible material,
- intensive stirring of the molten drop by a magnetic rf-field and rapid solidification guarantee a good homogeneity of the alloy,
- the process is quantitative and reproducible,
- the procedure is quick,

but

- very expensive equipment required,
- and controlling of the process is very difficult.

An overview of the methods applied for the preparation of radioactive targets was presented at the 2008 INTDS meeting [115]. Table 2 recapitulates the advantages and drawbacks of the methods commonly applied for radioactive targets preparation.

**Table 2:** Advantages and drawbacks of methods used for radioactive target preparation

Advantages	Drawbacks
*Electrospraying*	
High efficiency Simple equipment Homogeneity ~5 % (in some cases <2 %)	Thickness limitation, generally to few mg/cm^2^ Impurities from solvent Deposition of compounds only
*Electrodeposition*	
Process fast and simple, No cross contamination High efficiency Very good adherence Big range of the thickness, up to mg/cm^2^	Thickness homogeneity lower than by vac. evaporation Thickness limitation, generally <1 mg/cm^2^ Sometimes the deposits composition is unknown/uncertain Impurities from solvent
*Sedimentation*	
Very high efficiency Good for thick deposits	Limited to relatively thick deposits (mg/cm^2^) Impurities from solvent
*Electrophoresis*	
Quick Suitable for thick targets Deposits made of very fine grains what significantly influences the thickness homogeneity	Preparation of a colloidal suspension requires grinding of the powder (potential source of contamination) Upper thickness limitation (due to peeling off of the deposit) Problems caused by gas generated during the process
*High vacuum evaporation*	
High purity of the final product Relatively very high thickness homogeneity Suitable for very thin targets	Very low efficiency Time-consuming preparation of the material for evaporation
*Melting/alloying by levitation*	
Is very suitable for refractory metals No contact with crucible, no impurities	Requires a big amount of the evaporated or melted material Very expensive equipment

In cases when metallic targets are required it is necessary to conduct the material transformation, in most cases an oxide reduction. This can be performed using thorium or tantalum as reductants [116] applying similar procedures as described in Sect. "Conversion into metallic form". More attention has to be given when choosing the crucible to be used as a reduction vessel. For example the reduction of Pu oxide is more favourable to be performed in a vessel made of tantalum carbide, as Pu metal reacts with tantalum very easily. For some actinide materials with very high melting temperature (Cm, Cf) crucibles machined of vitreous carbon are recommended to eliminate a possible contamination originating from the crucible [117].

### Characterisation of the radioactive targets

The thickness is usually determined either by weighing or by alpha or gamma ray counting [118]. The last-mentioned methods are also used for the thickness homogeneity determination [48]. The thickness can as well be determined by non-contact devices based on optical profilometry.

The number of atoms in the target can be determined with high accuracy by destructive isotope dilution mass spectroscopy (IDMS) measuring the ratio of two isotopes of the same element (the second isotope is added to the sample in a very precisely known amount) [119].

## Stripper targets

The most commonly used stripper target is the carbon foil. Why? May be as D. Ramsay said, [120] wondering why so much work is done on extending the in beam life-time of carbon strippers: “Like people climb the mountains, because they are, the physicists use the carbon foils because we can make them”.

But there are some other reasons why the C-foils are still dominant. In case of heavy ions the energy of projectiles delivered by the accelerator with use of the C-foils as strippers is higher than that for gas strippers. In addition, the foils are easier to operate [121] but they suffer from short beam life-time. The extension of the life-time of carbon stripper foils was and still is a great concern for stripper users and makers. Various attempts such as foil slackening [122], graphitization [123], annealing, evaporating using various heating techniques [124] were undertaken to improve the C-foil life time. Although there are studies on non-carbon foil strippers (e.g. gaseous strippers [125]) especially for very intensive beams of heavy ions, it looks that carbon foils or their modifications will remain in service. There are intensive studies on strippers made of Diamond-Like Carbon DLC [126], nitrided carbon [127], HBC- hybrid boron–carbon [128].

Ramsay in his paper discussing alternative strippers ended with the conclusion: “May be one foil the size of a large pizza could be rotated like a phonograph record and would last 3 years” thus predicting a solution applied for example at RIKKEN for stripping uranium ions.

Large multilayer stripper foils, may be not as large as a pizza, were reported in several papers by Hasebe et al. [130] C-foils enhanced with polymer and oscillated during operational time displayed greatly improved life-time [129].

Carbon foils can be produced by various methods such as arc evaporation [131, 132], resistant heating [49], e-gun heating [76, 133] and laser ablation [134] but as well by cracking of organic compounds [135, 136] or applying the HIVIPP method [137, 138]. Application of the sputtering process [139] results in C-foils of much better quality, with higher stability and elasticity than those produced by evaporation. The life-time of stripper foils produced by sputtering can be up to 10 times higher than that of the evaporated ones [60] but method never become routinely used as it is very inefficient method (low deposition rate).

An extensive overview of methods for C-foil production was recently presented by Stoner [140] at the 22nd INTDS meeting in Gaithersburg.

## Gas targets

The term ‘gas target’ is used in an ambiguous way. Some uses the term referring to a target in the gaseous state while others mean the targets containing a gaseous element such as hydrogen, oxygen etc.

The simplest gaseous target is made of a gas container with an entrance window made of a thin foil. This window implies a restriction on the beam power to avoid rupture of the window or aging of the window material and thus, causing vacuum problems. Another important drawback of such targets is the energy straggling of the projectiles, especially in case of heavy ions. When passing through the entrance window they lose energy in a varying degree and thus the beam contains projectiles of various energy. These drawbacks can be overcome with windowless high-density gas targets using gas jets [141]. In case of studies carried with light projectiles of medium energy the use of thin polymer foils (thus consisting only of low-Z elements) as a window material can be considered [142, 143].

### Solid target of gaseous elements

Targets of gaseous elements can also be produced by implantation of gas atoms into a solid backing [144, 145] using the ions accelerated to an appropriate energy. This method was reported by a few target makers but never become widespread. The main pitfall of the method is the only semi-empirical knowledge of the implanted material deposition depth. The knowledge of this depth is important to make sure that the ions bombarding the target actually reach the deposit. The ‘thickness’ of the implanted material is another substantial aspect of target prepared this way. The studies by Almén and Bruce [146] on implantation depth for ions with an energy range suitable for implantation (keV) showed that the target material reaches saturation already at the level of a few μg/cm^2^. For most of the accelerator-based studies this means an insufficient number of atoms to obtain meaningful statistics. Another important drawback of this method is the stability of the deposits as the implanted ions migrate back to the surface and escape.

An alternative method used to prepare gaseous targets is based on the ability of certain metals (Ti, Er) to sorb the gas. It is applied to produce targets such as hydrogen and its isotopes [147–149].

Solid target of gaseous elements can as well be produced as compounds such as oxides [150], hydroxides, fluorides and also organic ones such as deuterated polyethylene used to produce deuterium targets [151] or tristearin to produce the targets with a high stable content of hydrogen [152].

Very often targets made now a days of the gaseous materials are in solid phase as the cryogenic technology is applied for their production, for example by cooling the gas container with liquid nitrogen [153] or by dropping jets of droplets of the frozen hydrogen or deuterium [154] to be introduced into the beam path [155, 156].

## Heat dissipation

A very important but not yet solved issue in target use and thus in target preparation is the dissipation of the heat deposited in the target by the bombarding projectiles.

$$ W = dE \times I $$

where *W* is the power deposited, *dE* the energy absorbed in the target (depending on the stopping power of the target material for the projectile of given energy) and *I* is the beam intensity.

The amount of the power deposited in the target influences the target life-time. Generally the heat can be dissipated by conductivity, radiation and convection. Taking into account that targets are relatively very thin (what in consequence means low cross-sectional area) and are exposed to the projectiles in vacuum the dissipation of the deposited heat is problematic.

**Fig. 18:**
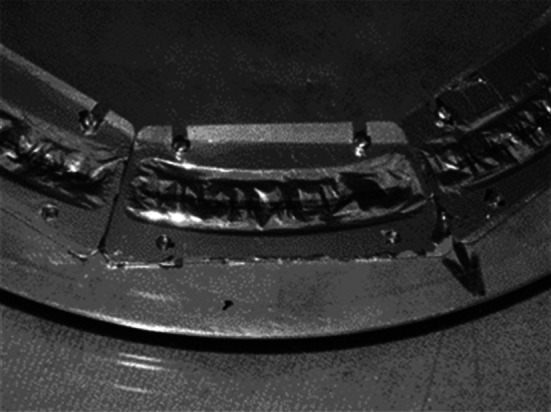
Target wheel used at Argonne lab for SHE project (SHE stands for Super Heavy Element) [www.phy.anl.gov/targetlab/gallery_files/pg18.html]

To extend the target life-time many experiments use a rotating target wheel (Fig. 18) composed of several targets and thus causing the heat to be deposited to a much bigger area than in case of a target mounted at a fixed position. This solution assures not only the distribution of the deposited power along the larger target area but as well gives the heat a time to dissipate, at least partially, from the target.

The first ‘wheel’ was applied already in 1932/1933 in experiments of elements transmutation by protons carried out by Oliphant and the Rutherford’s team: “The beam falls upon the target. This is of steel and has three faces at 45° to the axis, [...]. The target is carried on a water-cooled stem which rotates in a ground joint and can be set so as to bring any one of the three faces into a beam” [5].

Application of a rotating wheel of 10 cm diameter with eight banana-shaped carbon strippers applied in the beam line in Argonne at tandem accelerator extended the foils lifetimes by a factor of 12 as reported by G.E. Thomas [157].

In case of targets other than carbon a thin layer of carbon can be added to the target to improve the heat loss by radiation. The combination of both solutions—a target wheel [158] containing Pb foils covered on both sides with a thin layer of carbon (carbon emissivity *ε*_C_ = 0.8)—was used for studies with high intensity heavy ion beams at GSI [159].

Further, enlarging of the wheel diameter and blackening the wheel material to enhance the heat dissipation by radiation significantly increased the targets lifetime under bombardment with a high intensity beam (applied at GSI for SHIP studies [160]). A gas-cooled wheel with 16 sandwich targets (C/metal/C) applied at experiments searching for super heavy elements performed at RIKEN [161] survived 5 days of irradiation with ^70^Zn ions of typical intensity of 3.2 × 10^12^ s^−1^. Compared to the target life-time at experiments from 1988 [162] this means an improvement by a factor of 10.

Further studies on extending the target life-time or on measurement non-interrupted by target changing are carried on. The bench of water-cooled rotating targets proposed by Yoshida et al. [163] is a system composed of a chamber with the target disks and a disk-changing system allowing automatic replacement of the target wheel without opening the beam line thus, without interrupting the experiment.

Other possibilities of extending the target life-time are related to beam wobbling or intensive holder cooling, but all of these methods are not sufficient to keep a target cold under an intensive beam. So there is still a lot to be done in this area.

## Targets for radioisotopes production

Targets used for production of medical radioisotopes in reactions with projectiles provided by accelerators are usually very thick comparing to targets used in nuclear physics studies. A high thickness of the targets is required to assure the best possible yield of isotopes production but as well they have to be very robust to sustain the energy deposited in the target by very intensive beams. On the other hand they should allow easy and efficient extraction of the produced isotope, often very quickly due to its short half-life in the range of hours or even minutes.

Different requirements from the ‘medical’ target and another characteristic cause other problems encountered at their preparation, than discussed above. The discussion of these problems is beyond this review. Many detailed information related to these problems can be found in proceedings of Workshops on Targetry and Target Chemistry (WTTC), meetings initiated in 1985 [164].
